# Heterostrain-enabled dynamically tunable moiré superlattice in twisted bilayer graphene

**DOI:** 10.1038/s41598-021-00757-x

**Published:** 2021-11-01

**Authors:** Xuejiao Gao, Hao Sun, Dong-Ho Kang, Chongwu Wang, Qi Jie Wang, Donguk Nam

**Affiliations:** School of Electrical and Electronic Engineering, Nanyang Technological University, 50 Nanyang Avenue, Singapore, 639798 Singapore

**Keywords:** Materials science, Nanoscience and technology

## Abstract

The ability to precisely control moiré patterns in two-dimensional materials has enabled the realization of unprecedented physical phenomena including Mott insulators, unconventional superconductivity, and quantum emission. Along with the twist angle, the application of independent strain in each layer of stacked two-dimensional materials—termed heterostrain—has become a powerful means to manipulate the moiré potential landscapes. Recent experimental studies have demonstrated the possibility of continuously tuning the twist angle and the resulting physical properties. However, the dynamic control of heterostrain that allows the on-demand manipulation of moiré superlattices has yet to be experimentally realized. Here, by harnessing the weak interlayer van der Waals bonding in twisted bilayer graphene devices, we demonstrate the realization of dynamically tunable heterostrain of up to 1.3%. Polarization-resolved Raman spectroscopy confirmed the existence of substantial heterostrain by presenting triple G peaks arising from the independently strained graphene layers. Theoretical calculations revealed that the distorted moiré patterns via heterostrain can significantly alter the electronic structure of twisted bilayer graphene, allowing the emergence of multiple absorption peaks ranging from near-infrared to visible spectral ranges. Our experimental demonstration presents a new degree of freedom towards the dynamic modulation of moiré superlattices, holding the promise to unveil unprecedented physics and applications of stacked two-dimensional materials.

## Introduction

The formation of moiré superlattices in twisted layers of two-dimensional (2D) materials has recently become a crucial method to realize unprecedented photonic and electronic properties that are unattainable in the constituent layers^1–6^. For instance, twisted bilayer graphene (TBG) with a magic twist angle can flatten the electronic band structure near zero Fermi energy, which allows achieving Mott insulating behavior and unconventional superconductivity^1,2^. Besides, twisted transition metal dichalcogenide (TMD) heterobilayers have enabled trapping interlayer excitons in the potential wells created by moiré superlattices^3–6^.

Very recently, heterostrain in twisted layers of 2D materials has arisen as a powerful, new degree of freedom to tailor moiré patterns and the resulting physic properties of the stacked 2D materials^7–13^. Heterostrain is inequivalent strains on the two constituting layers of stacked 2D materials, which leads to the slippage of one layer over the other due to inequivalent lattice deformation^14–18^. Theory results have recently predicted that heterostrain can modulate the local atomic registry of moiré superlattices and enable intriguing properties such as flat moiré bands in TBG^12^, topologically protected helical modes in twisted TMD^19^, and excitonic instability in TBG^13^. The effect of heterostrain is expected to be far more significant than that of homostrain on altering moiré patterns^7^, which may enable discovering a new class of physical properties beyond what have thus far been observed in the absence of heterostrain^12,13^. For instance, heterostrain-enabled one-dimensional (1D) moiré potentials have shown linearly polarized photoluminescence emission with two orders of magnitude higher intensity^20^.

Overall, the desired physical properties of moiré superlattices are very sensitive to the modulation of the moiré potential landscape^21,22^, which requires the precise control of the twist angle and heterostrain during device fabrications. Recent experimental studies have demonstrated the continuous tuning of the twist angle, which substantially alleviates the strict requirement for controlling the twist angle during the fabrication^21–23^. However, controlling heterostrain dynamically has yet to be experimentally realized, limiting the feasibility of heterostrain-enabled on-demand manipulation of moiré superlattices.

Here, we present a generalized method for dynamically tuning heterostrain in TBG to modify moiré superlattices. TBG devices were assembled on a flexible PMMA-coated PET substrate by mechanical stacking. A relatively large twist angle of 13.2° was intentionally used to minimize the interlayer friction force^14–16^, which played a key role in achieving a large heterostrain of up to ~ 1.3%. The induced heterostrain in TBG devices was dynamically tunable by continuously changing the bending radius of the PET substrate. Raman spectroscopy provided unambiguous evidence for the generation of a substantial heterostrain by presenting triple G peaks that arise from both unstrained and strained graphene layers having a single degenerate mode of the G peak and two split G^+^ and G^−^ peaks, respectively. Strong polarization dependence of the triple G peaks further confirmed the formation of heterostrain. We also verified the influence of the experimentally obtained heterostrain on the modulation of moiré superlattices and the electronic band structures of TBG through tight-binding simulations. The 1.3% heterostrain was predicted to allow achieving multiple absorption peaks including one in the near-infrared spectral range that is attributed to the optical transition between the two saddle points, which is forbidden in the absence of heterostrain.

## Results

### Device configuration of the TBG device with dynamically tunable heterostrain

**Figure 1:**
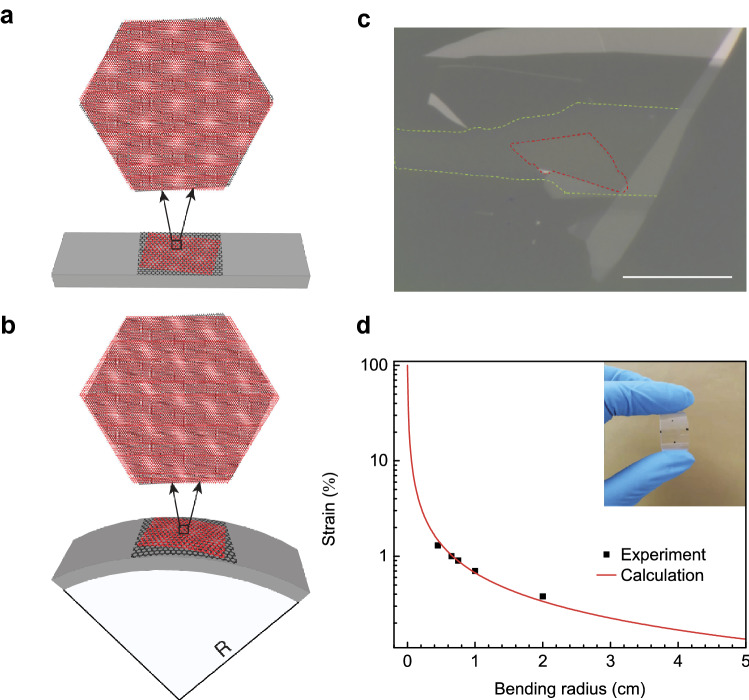
Heterostrain-enabled dynamically tunable moiré superlattice in TBG. (**a**, **b**) Schematic illustration of dynamically tunable moiré superlattices in TBG on a flexible substrate before and after bending. (**c**) Optical microscope image of the TBG device with a 13.2° twist angle used in our study. Scale bar, 20 μm. (**d**) Calculated (red line) strain on PET surface and experimental (black squares) uniaxial strain in bottom graphene. Thickness of our PET substrate is 135 μm. Inset: photo showing the flexibility of our substrate with the TBG device on top.

Figure 1a,b present schematic illustrations of our typical device configuration that allows the dynamic control of the heterostrain in TBG. First, graphene flakes were exfoliated and deposited onto a silicon (Si) substrate with a 285-nm silicon dioxide (SiO_2_) layer to allow convenient identification of monolayer graphene. The number of graphene layers was identified via various methods including Raman spectroscopy and optical contrast (see Supplementary Note 1 for more details on the sample identification). The crystal orientation was determined from the cleaved angles of exfoliated flakes^24^. The cleaved edge was aligned to the long side of a PMMA-coated PET substrate on a transfer stage.

First a large graphene layer on the SiO_2_/Si substrate (e.g., Fig. S1b) was picked up with PMMA-coated PET substrate, which we call bottom graphene. Then the entire stack consisting of graphene, PMMA, and PET substrate was aligned to and picked up a relatively smaller second graphene layer (Fig. S1a; top graphene) with a twist angle of 13.2° between the first and second layers, completing the fabrication process of TBG on a flexible substrate (see Supplementary Note 2 for more details on the fabrication procedure). As shown in Fig. 1c, bottom graphene thoroughly separates the top graphene and PMMA-coated PET, such that strain from PET will only be transferred to the bottom graphene. Upon bending the PMMA-coated PET substrate, the bottom graphene layer that is strongly adhered to PMMA is uniaxially stretched whereas the top graphene layer undergoes a negligible amount of lattice deformation owing to the weak van der Waals bonding between the two graphene layers^14–16^, thus enabling the dynamic modulation of heterostrain. As a result, the moiré pattern in TBG can be strongly modified as shown in Fig. 1a,b.

Figure 1c presents an optical microscope image of the TBG device used in our study. For bending apparatus (Supplementary Note 3), bending radius ($R$) and substrate thickness ($t$) together determine the strain generated on PET surface, following the relationship of $\varepsilon \approx t/2R$ ^25^ (Fig. 1d). As the bending radius decreases gradually, the uniaxial strain applied at the top surface of the substrate increases as shown in the red solid line in Fig. 1d. Strain in graphene was extracted from Raman shift of G (G^−^ peak when G peak is split) by ${\varepsilon }_{r}=\left({\lambda }_{r}-{\lambda }_{r=0}\right)/a$, where ${\lambda }_{r}$ is the G peak position at bending radius $r$, ${\lambda }_{0}$ is the G peak position at bending radius of 0 cm, and $a=31.7$ cm^−1^/% is the strain coefficient. Table S1 summaries the G peak position, red shift and derived strain. Strain values measured on the bottom graphene layer on the bent substrate with various bending radii (black squares) agree well with the calculated values, confirming the successful strain transfer from the substrate surface to the bottom graphene layer owing to the strong adhesion between bottom graphene and PMMA (see Supplementary Note 3 for strain measurement via Raman spectroscopy). The inset of Fig. 1d displays the bent substrate with TBG on top.

### Experimental verification of dynamically tunable heterostrain in TBG

**Figure 2:**
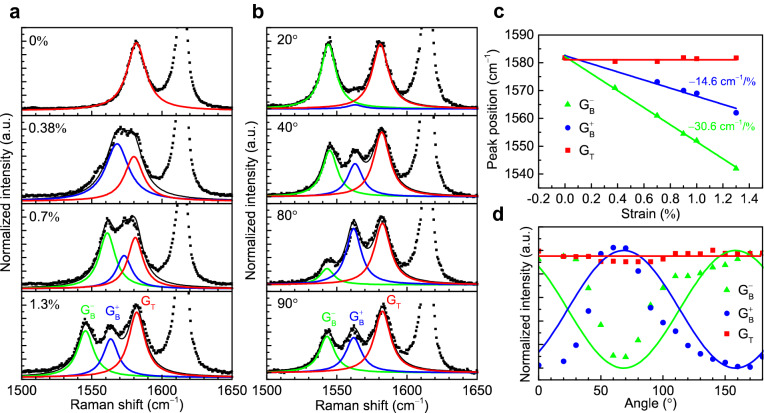
Verification of heterostrain in the TBG device via Raman spectroscopy. (**a**) Raman spectra measured with different bending radii, showing triple peaks arising from the heterostrained TBG device. (**b**) Polarization-resolved Raman spectra showing the intensity-modulated G and G peaks and the polarization-insensitive G_T_ peak. Uniaxial strain of 1.3% was applied to bottom graphene. (**c**) The shift of the three G peaks arising from strained bottom and unstrained top graphene layers. (**c**) The intensity of the three G peaks as a function of the rotational angle. Green curve: ${I}_{{G}_{B}^{-}}\propto {sin}^{2}\left(\theta -{34.2}^{\circ }\right)$; Blue curve: ${I}_{{G}_{B}^{+}}\propto {cos}^{2}\left(\theta -{34.2}^{\circ }\right)$.

**Figure 3:**
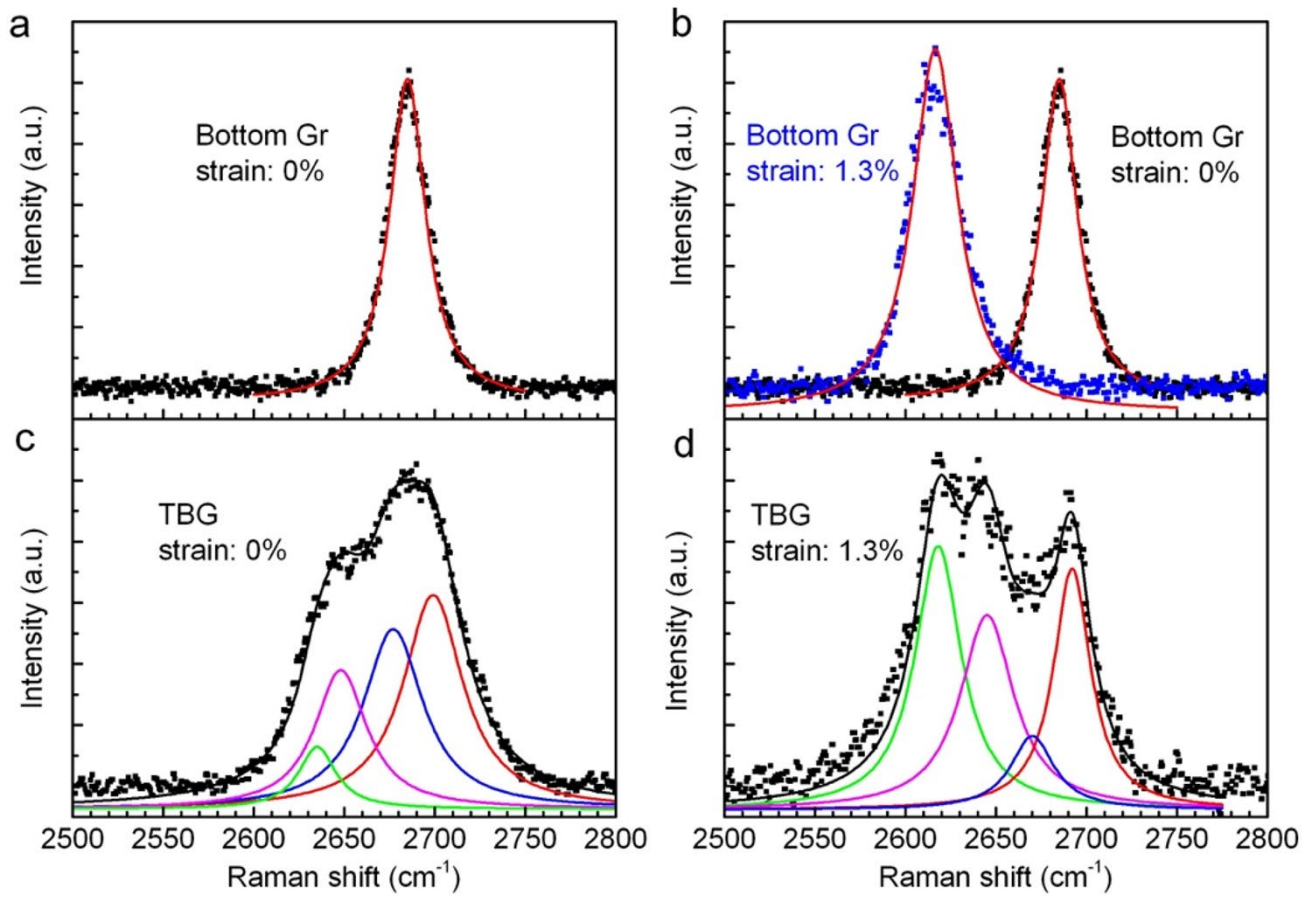
Raman spectra of graphene 2D peak give evidence of weak electronic coupling in TBG and heterostrained TBG. (**a**) Raman spectrum of 2D peak in unstrained bottom graphene. (**b**) Raman spectrum of 2D peak in bottom graphene under 1.3% uniaxial strain (blue dots). 2D spectrum of unstrained graphene was added for reference. (**c**) Raman spectrum of 2D peak in unstrained TBG. (**d**) Raman spectrum of 2D peak in TBG under 1.3% heterostrain. Dots are experimental data and curves are obtained by Lorentz fitting.

Raman spectroscopy was used to verify the existence of dynamically tunable heterostrain in TBG. Figure 2a presents Raman spectra measured for TBG on the flexible substrate with different bending radii. The amount of bending and the corresponding strain induced in the bottom graphene layer increases from the top to the bottom panels, with the topmost panel for no bending and 0% strain. The G peak of unstrained TBG shown in the topmost panel appears at 1582 cm^−1^ with the full width at half maximum (FWHM) of 16 cm^−1^ (Fig. 2a), consistent with the unstrained graphene in literature^26^. The red curve shows a fitted Lorentzian function. Graphene G peak corresponds to in-plane optical mode E_2g_, which however reflects no information about interlayer coupling. Graphene 2D peak is later studied in Fig. 3 to distinguish our weakly coupled TBG from decoupled bilayer graphene. A strong peak at 1615 cm^−1^ arises from the PET substrate^27^.

Interestingly, the substrate bending causes the single G peak to split into multiple peaks that are clearly discernable as shown in the bottom three panels. At a bending radius of 2 cm (the second panel from the top, Fig. 2a), the center position of the G peak shifts to 1567 cm^−1^, and the FWHM becomes wider to 30.5 cm^−1^. This seemingly single peak can be well fitted by two subpeaks at 1582 cm^−1^ and 1571 cm^−1^. The blue curve at 1571 cm^−1^ can be attributed to the bottom graphene layer under uniaxial strain and is termed G_B_. The strong bonding between the bottom graphene layer and PMMA leads to the induced strain at the PMMA surface to be successfully transferred to the bottom graphene layer. This bending-induced strain in graphene has already been confirmed by Ferrari et al.^25^ The strain value of 0.38% is derived by using the strain-shift coefficient of − 31.7 cm^−1^/%. It has recently been theoretically and experimentally studied that the interlayer friction between two van der Waals layers can be significantly minimized when the interlayer stacking is incommensurate^14–16^. At a twist angle of 13.2°, the vanishingly small interlayer friction allows the top graphene layer in our TBG device to be uninfluenced by the lattice distortion of the underlying bottom graphene layer, thus producing a Raman signal at 1582 cm^−1^ that is the same as the unstrained case. We term this peak G_T_ since it is from the top graphene layer. At larger bending radii, the peak from the G_B_ peak from the bottom strained graphene layer further splits into two peaks consisting of the G (blue) and G (green) peaks as shown in the bottom two panels of Fig. 2a. G and G peaks correspond to the doubly degenerate ${\mathrm{E}}_{2\mathrm{g}}^{+}$ and ${\mathrm{E}}_{2\mathrm{g}}^{-}$ phonons, which are parallel and perpendicular to the applied uniaxial strain, respectively. The G_T_ peak remains unchanged while the G peak shifts by ~ 37 cm^−1^, which provides strong evidence for the development of substantial heterostrain in the TBG device. The measured peak positions of the three peaks, G_T_, G and G, at different bending radii are summarized in Fig. 2c.

To further provide the evidence of the heterostrain in our TBG device, we performed polarization-dependent Raman measurements. The laser emission was linearly polarized and the uniaxially strained device was rotated continuously while the bending radius was fixed. As shown in Fig. 2b, the intensities of the G and G peaks are modulated as the angle between the sample orientation and the laser polarization direction is changed. In contrast, the intensity of the G_T_ peak remains largely constant. Figure 2d summarizes the Raman response from uniaxially strained graphene as function of the rotational angle, showing the clearly opposing trends between the G and G peaks. This polarization dependence study reconfirms that the G and G peaks are from the uniaxially strained bottom graphene layer^25^ and that the G_T_ peak is from the unstrained top graphene layer, which results in the heterostrained TBG device.

Interfacial electronic coupling plays a critical role for unprecedented physical properties in moiré materials, therefore it is necessary to distinguish our heterostrained TBG from decoupled bilayer graphene. In Fig. 3, Raman 2D peaks of bottom graphene and heterostrained TBG are investigated as a reference for interlayer coupling effect. For unstrained bottom graphene, a symmetric peak is fitted by a single Lorentzian at 2685 cm^−1^ with FWHM of 24 cm^−1^ (Fig. 3a). However, an unsymmetric 2D peak with FWHM of 84 cm^−1^ is measured on unstrained TBG and fitted by four Lorentzians (Fig. 3c), which reflects different doubling resonant processes in electronically coupled bilayer system. When bottom graphene is under 1.3% uniaxial strain, the 2D peak fitted by a single Lorentzian red-shifts to 2616 cm^−1^ (Fig. 3b). For TBG under 1.3% heterostrain, 2D peak is unsymmetric and fitted by four Lorentzians, more complex than a single superposition of 2D peaks on monolayer graphene flakes under 0% and 1.3% uniaxial strain (Fig. 3b). Therefore, we conclude that a weak interlayer coupling effect still exists when 1.3% heterostrain is applied on TBG with 13.2° misorientation.

### Modified band structures and optical responses in heterostrained TBG

**Figure 4:**
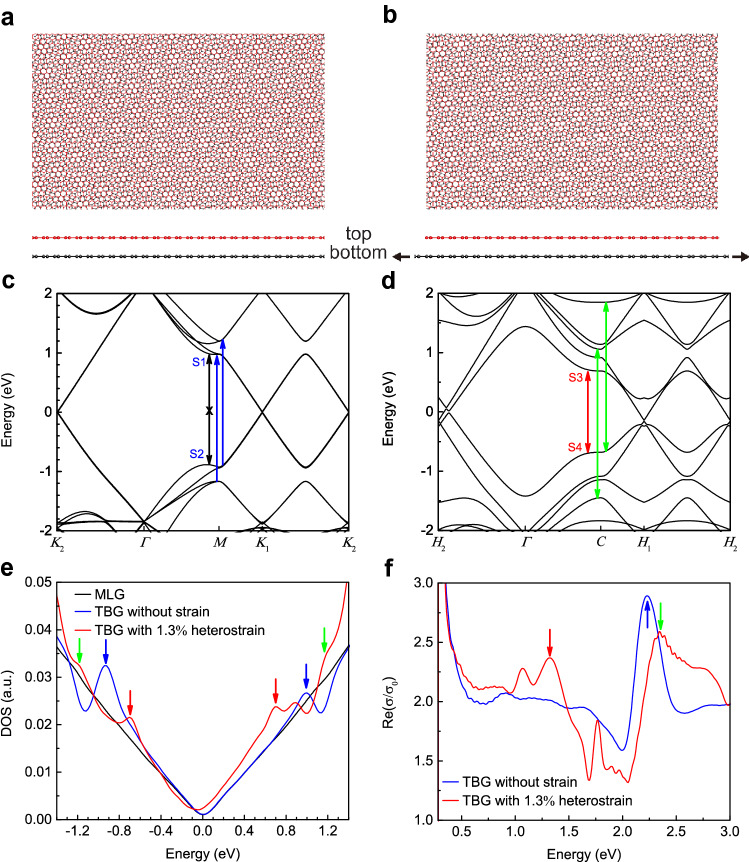
Tight-binding simulations of TBG with and without heterostrain. (**a**, **b**) Moiré patterns of TBG with 0% and 1.3% heterostrain. (**c**, **d**) Band structure of TBG with 0% and 1.3% heterostrain. (**e**) DOS of pristine graphene (black), TBG (blue) and TBG under 1.3% heterostrain (red). (**f**) Dynamic conductivity spectra of TBG with (red) and without (blue) heterostrain, which are normalized to pristine graphene.

The existence of heterostrain is expected to strongly modify the moiré potential landscapes, which influence the electronic band structures and optical properties of the TBG devices. Figure 4a,b show the moiré superlattices of TBG without and with heterostrain, respectively. The twist angle is set to 13.2° for both Fig. 4a,b and the applied heterostrain in Fig. 4b is 1.3% to reflect the experimental details used in our study. TBG in the absence of heterostrain presents a typical moiré superlattice in hexagonal pattern (Fig. 4a) whereas the applied heterostrain can distort the moiré superlattice into oblique pattern (Fig. 4b). Figure 4c,d display the electronic band structures for Fig. 4a,b, respectively, that were calculated by using tight-binding simulations^28,29^. The calculation details are provided in Supplementary Note 4. The presence of 1.3% heterostrain in the TBG device significantly modifies the electronic band structures as can be clearly seen by comparing Fig. 4c,d.

The changed electronic band structures in the presence of heterostrain result in the altered density of states (DOS) (Fig. 4e), which ultimately have a profound impact on the optical properties of the TBG (Fig. 4f). Figure 4e presents the DOS spectra for TBG with (red curve) and without (blue curve) heterostrain. The DOS spectrum of monolayer graphene (MLG) is also presented as comparison (black curve). Compared to MLG, both TBG cases show pronounced DOS peaks. TBG without heterostrain shows a pair of DOS peaks (marked as blue arrows), one in valence band and another in conduction band, which are enhanced over MLG. The pronounced DOS peaks in unstrained TBG can be ascribed to the flatten dispersion curves marked as S1 and S2 in Fig. 4c, which are the saddle points^28^. The 1.3% heterostrain reduces the energy of the two saddle points, shifting the two DOS peaks towards zero (marked as red arrows). In addition, heterostrain increases the number of pronounced DOS peaks (marked as red and green arrows). These new DOS peaks can be attributed to the strongly modified electronic band structures under the influence of heterostrain as shown in Fig. 4d. The colors of the arrows in Fig. 4e for heterostrained TBG are matched to the colors of optical transition labelled in Fig. 4d,f.

We also calculated the real part (Re) of the dynamical conductivity, $\sigma$, of unstrained and heterostrained TBG devices (Fig. 4f). The spectra are normalized to the conductivity of monolayer graphene (MLG), ${\sigma }_{0}$*.* The calculated Re (${\sigma /\sigma }_{0})$ is equivalent to the relative enhancement of the optical absorption in TBG compared to MLG. The optical absorption in two layers of graphene is 2 ${\sigma }_{0}$ that is ~ 4.6%^30^. Unstrained TBG (blue curve) shows strongly enhanced optical absorption (marked as blue arrow in Fig. 4f) over 2 ${\sigma }_{0}$ at ~ 2.2 eV mainly due to the pronounced DOS at saddle points (marked as S1 and S2 in Fig. 4c). Optical transition between S1 and S2 are not allowed in TBG, because the matrix element is calculated to be zero due to the symmetry protection^28^. Interestingly, heterostrained TBG (red curve) possesses multiple peaks enhanced over 2 ${\sigma }_{0}$ (marked as red and green arrows in Fig. 4f), which are also largely owing to the pronounced DOS peaks under the influence of heterostrain (marked as red and green arrows in Fig. 4e). In addition, optical transition between S3 and S4 in Fig. 4d becomes optically active (i.e., the matrix element is non-zero due to symmetry breaking, see the Supplementary Note 7 for more details) in 1.3% heterostrained TBG, which allows an enhanced optical absorption at the technologically important near-infrared spectral range.

## Discussion

In summary, we demonstrated a strain engineering platform that enables the dynamic tuning of heterostrain in TBG. Raman spectroscopy measurements confirmed the existence of heterostrain by showing triple G peaks; the unstrained top graphene layer showed the G peak that is the same as in the pristine MLG whereas the strained bottom graphene layer presented the two split G peaks, which also showed strong polarization dependence. It was verified that the heterostrained TBG possesses distorted moiré superlattices, which strongly influence the electronic band structures and optical properties. Our heterostrain platform gives a new approach to tune the moiré materials continuously and accurately, thus may find important applications in tunable electronics and optoelectronics based on moiré-engineered graphene. This approach is very general and can be employed for modulating moiré superlattices in twisted transition metal dichalcogenide (TMD) heterostructures as well, which are expected to play important roles in engineering moiré excitons for a variety of applications including quantum emission^3–6^. Moreover, the dynamically tunable moiré superlattices enabled by heterostrain may achieve unprecedented physical properties. It is also worth noting that such heterostrain in TBG can also be achieved in an integrated strain engineering platform on a silicon substrate^31,32^.

## Methods

### Sample preparation

Graphene flakes were prepared by mechanical exfoliation of Kish graphite (Natural Kish graphite, grade 300, Graphene Supermarket) by scotch tape and deposited onto Si/SiO_2_ substrate (285 nm SiO_2_ for good optical contrast). Number of graphene layers was identified by analyzing optical contrast and Raman spectra. Long graphene strips were selected as bottom layers of TBG, while smaller monolayer flakes were selected as tops layers. Both the selected bottom and top layers has straight edges, such that crystal orientation could be identified from flake edges.

A 135-μm thick PET substrate was cut into 1 cm × 5 cm rectangles. PET substrates were cleaned by acetone, isopropanol alcohol (IPA) and deionized (DI) water, and then dried at 65 °C for 5 min. About 400 nm polymethyl methacrylate (PMMA, 950 PMMA A6, MICROCHEM) was spin-coated onto PET at 4500 rpm for 90 s.

### Mechanical stacking of twisted 2D materials

Twisted bilayer graphene was mechanically stacked on PMMA-coated PET with a home-made transfer stage. First, long edge of bottom graphene was aligned along horizontal direction by rotating the sample stage. At the same time, PMMA-coated PET thin film was attached to a PDMS stamp of ~ 4 mm × 2 mm × 0.6 mm, which was adhered to a glass slide. Bending direction of PET rectangle was also aligned to horizontal direction, such that uniaxial strain will be applied along long side of bottom graphene. Then Glass/PDMS/PET/PMMA stack was used to pick up the bottom graphene layer. After baking at 180 for 2 min, bottom graphene is firmly adhered to PET substrate. Next top graphene was aligned 13.2° to horizontal, which was subsequently picked up by Glass/PDMS/PET/PMMA/Bottom graphene. Finally, detach PET substrate from glass slide and PET/PMMA/TBG was obtained. Cured PMMA can successfully transfer strain from PET to bottom layer of TBG during the bending process.

### Raman measurements

Graphene G peaks were measured by Raman spectroscopy (Alpha300 M+, WITec) with a 100× objective and 1800 g/mm grating. Laser wavelength was 532 nm. Before measurement, laser power was adjusted to low level to avoid heating effect. Lorentz fitting was applied to determine peak position and FWHM.

## Supplementary Information

Supplementary Information.
