# Supplementary material for: Heterostrain-enabled dynamically tunable moiré superlattice in twisted bilayer graphene
Source: Sci Rep. 2021 Nov 1;11:21402. doi: 10.1038/s41598-021-00757-x (PMC8560801; doi:10.1038/s41598-021-00757-x)
Supplement: Supplementary file 1 — Supplementary Information. [file 41598_2021_757_MOESM1_ESM.pdf]

Supporting information for

Heterostrain-enabled dynamically tunable moiré superlattice in twisted bilayer graphene

Xuejiao Gao[1†], Hao Sun[1†], Dong-Ho Kang[1], Chongwu Wang[1], Qi Jie Wang[1] and Donguk Nam[1*]

[1]School of Electrical and Electronic Engineering, Nanyang Technological University, 50 Nanyang Avenue, Singapore 639798, Singapore

[†]These authors contributed equally to this work.

[*]E-mail: dnam@ntu.edu.sg

Supplementary Note 1: Identification of monolayer graphene

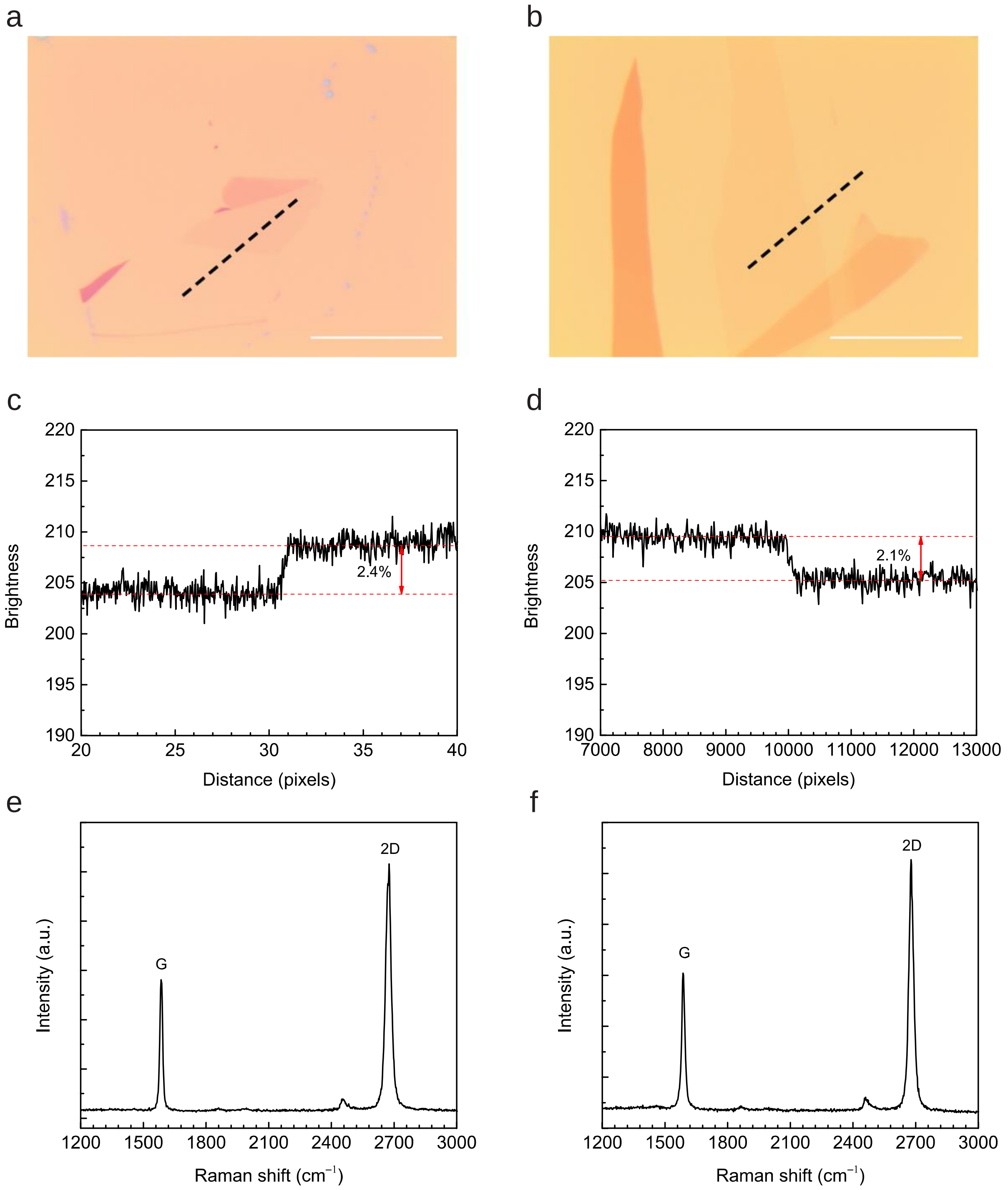

Figure S1 | Identification of monolayer graphene on SiO_2/Si substrate. a and b, optical images of monolayer graphene. Scale bar, 20 μm. **c and d**, optical contrast of graphene along the dashed

lines in Figs. S1a and S1b, respectively. **e and f**, Raman spectra of graphene in Figs. S1a and S1b, respectively.

Figures S1a and S1b show optical images of two flakes of monolayer graphene, which were transferred onto a PET substrate as top and bottom layers of TBG. Number of graphene layers was identified by analyzing optical contrast and Raman spectra. Optical contrast was defined as $1 - I_G/I_S$, where I_G and I_S are the brightness of graphene and substrate area in optical images. Brightness of an optical image can be extracted by using the equation $I = (333 \times R + 500 \times G + 166.6 \times B)/1000$,[1,2] where R, G, B present the red, green and blue element, respectively. Figures S1c and S1d show optical contrast of 2.4% and 2.1%, consistent with 2.3% optical absorption of monolayer graphene.[3] In the two Raman spectra shown in Figs. S1e and S1f, $I_{2D}/I_G \approx 2$, reconfirming the graphene to be monolayer.[4] Crystal orientation was identified from flake edges.[5]

Supplementary Note 2: Mechanical stacking of twisted 2D materials

A 135-μm thick PET substrate was cut into 1 cm × 5 cm strips. PET substrates were cleaned by acetone, isopropanol alcohol (IPA) and deionized (DI) water, and then dried at 65 ºC for 5 min. About 400 nm polymethyl methacrylate (PMMA, 950 PMMA A6, MICROCHEM) was spin-coated onto PET at 4500 rpm for 90 sec. Twisted bilayer graphene was mechanically stacked on PMMA-coated PET with a home-made transfer stage, following the steps shown in Fig. S2.

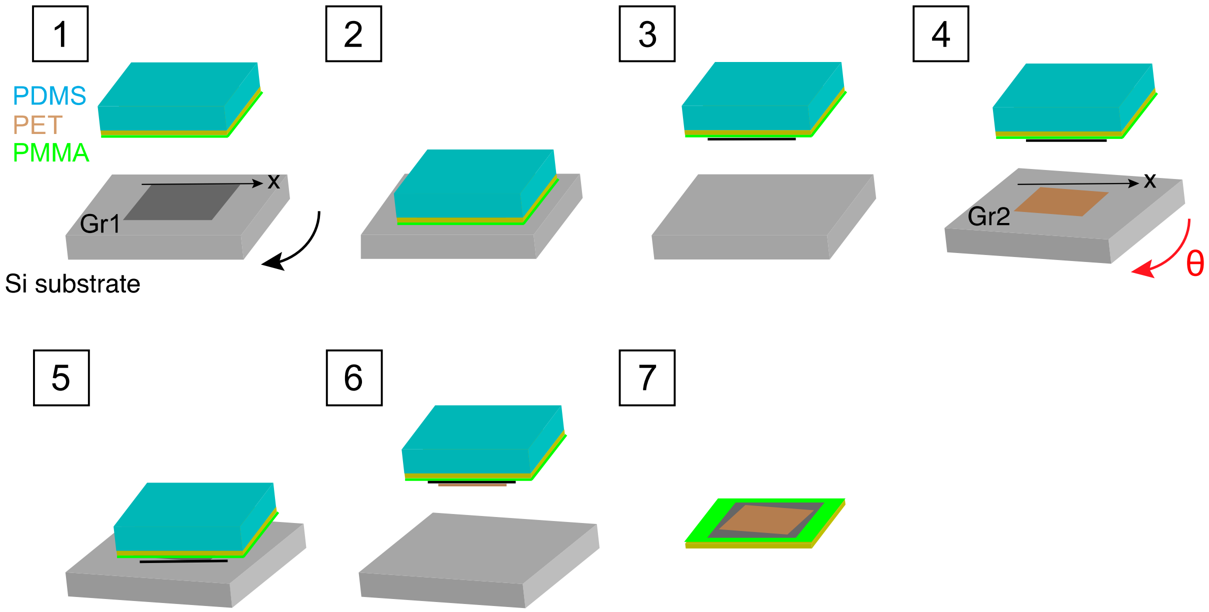

Figure S2 | Fabrication process of heterostrain-engineered TBG. 1, Long side of the first graphene (Gr1) is aligned along x direction by rotating the stage. PMMA-coated PET is attached to a PDMS stamp. **2,** Press PMMA/PET/PDMS onto Gr1. **3,** Pick up Gr1. And then bake Gr1/PMMA/PET at 180°C for 2 min, which firmly adhere Gr1 onto PET substrate. **4,** After cooling down, attach Gr1/PMMA/PET back to the PDMS stamp. Long side of second graphene (Gr2) is aligned along x direction. After alignment, rotate the Gr2 by θ (13.2°). **5,** Press Gr1/PMMA/PET/PDMS onto Gr2. **6,** Pick up Gr2. **7,** TBG with 13.2° is obtained with bottom layer adhered onto PMMA-coated PET substrate.

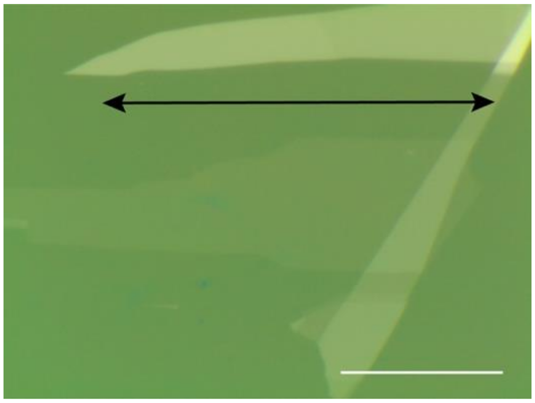

Figure S3 | Optical image of monolayer graphene. The image shows a monolayer graphene layer that has been successfully transferred onto a PMMA-coated PET substrate with long side aligned to bending direction as highlighted by the black arrow. Scale bar is 20 μm.

Supplementary Note 3: Heterostrain measurements by Raman spectroscopy

After stacking TBG on PMMA-coated PET, the flexible PET substrate with TBG on top was bended and fixed on a glass slide. Strain applied on graphene was tuned by changing the bending radius (Fig. S4). Graphene G peaks were measured by Raman spectroscopy (Alpha300 M+, WITec) with a 100× objective and 1800 g/mm grating. Laser wavelength was 532 nm. Before measurement, laser power was adjusted to low level to avoid heating effect. Lorentz fitting was applied to determine peak position and FWHM.

At each bending radius which was measured from the PET arch, strain in graphene was extracted from Raman shift of G (G^- peak when G peak is split), using the strain coefficient of 31.7 cm^{-1}/%. Table 1 summaries G peak position, red shift and derived strain.

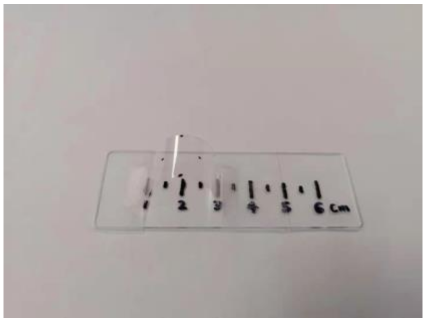

Figure S4 | Bending apparatus. Bending radius is tuned by changing the distance between two ends of PET strip.

Table S1. Raman shift and strain on stretched graphene.

Bending radius (cm)	G or G^- peak position (cm^{-1})	Red shift $\Delta\lambda$ (cm^{-1})	Strain (%)
0	1582.0	0	0
2	1570.0	12.0	0.38
1	1560.0	22.0	0.7
0.75	1553.5	28.5	0.9
0.65	1551.0	31.0	1
0.45	1541.0	41.0	1.3

Supplementary Note 4: Simulation on band structure of heterostrained TBG

To calculate the energy spectrum of TBG with the heterostrain, the single-orbital tight-binding model was used, which can be expressed as:[6]

$$H_S = \sum_i t_{ij}(\vec{r}_{ij}) c_j^\dagger c_i + h.c. \tag{1}$$

where the hopping integral $t_{ij}(\vec{r}_{ij})$ between any of two carbon atoms with distance $\vec{r}_{ij} = (x, y, z)$ described by a function:[6]

$$t_{ij}(\vec{r}_{ij}) = t_0 \exp\left(-\beta\left(\frac{r_{ij}}{a_0} - 1\right)\right)\left(\frac{x^2+y^2}{r_{ij}^2}\right) + t_1 \exp\left(-\beta\left(\frac{r_{ij}-d}{a_0}\right)\right)\frac{z^2}{r_{ij}^2} \tag{2}$$

where $t_0 = -2.7\ eV$ is the nearest intralayer coupling, $t_1 = 0.48\ eV$ is the interlayer coupling, $\beta = 3.3$ is a dimensionless exponential decay factor, $a_0 = 0.142\ nm$ is the length of carbon-carbon bond in a pristine monolayer, and $d_0 = 0.335\ nm$ is the interlayer spacing. Interlayer hoppings between atoms for $r_{ij} \leq 4a_0$ are considered, while the nearest-neighbor intralayer hoppings are also considered. We calculate the energy spectrum of TBG with and without heterostrain. The superlattice constants of TBG can be determined by the commensurate rotation:[7,8]

$$\cos(\theta_n) = \frac{3n^2+3n+\frac{1}{2}}{3n^2+3n+1} \tag{3}$$

where $n = 0,1,2\ ...$ we choose $n = 2$, and get the rotation angle $\theta_2 = 13.174\ °$, which matches the rotation angle of TBG in our experiment. Thus, we have the superlattice constant vectors:

$$\vec{t}_1 = 2\vec{a}_1 + 3\vec{a}_2 \tag{4}$$

$$\vec{t}_2 = -3\vec{a}_1 + 5\vec{a}_2 \tag{5}$$

where $\vec{a}_1$ and $\vec{a}_2$ are the lattice constant vectors of pristine graphene unit cell. $|\vec{t}_1| = |\vec{t}_2| = 1.072\ nm$. With the heterostrain, the superlattice structure will be distorted, the lattice constants

$|\vec{t}_1| = 1.081\ nm$, $|\vec{t}_2| = 1.072\ nm$, and the angle between $\vec{t}_1$ and $\vec{t}_2$ is 60.4 °. Lattice relaxation in TBG may alter the electronic band structure and related optical and electrical properties. Nam et al.[9] theoretically demonstrated that lattice relaxation only causes a significant difference when rotation angle is smaller than 2°, and the energy change is up to 20 meV, which is a very small energy modification compared to our large band width of 1.34 eV. Experimental analysis of lattice relaxation was performed by using the selected-area electron diffraction (SAED)[10], which showed that no appreciable lattice reconstruction occurs when rotation angle is larger than 4°. Therefore, atomic-scale reconstruction in our TBG with twist angle of 13.2° is very small and negligible. Our model and calculations were performed without considering lattice relaxation.

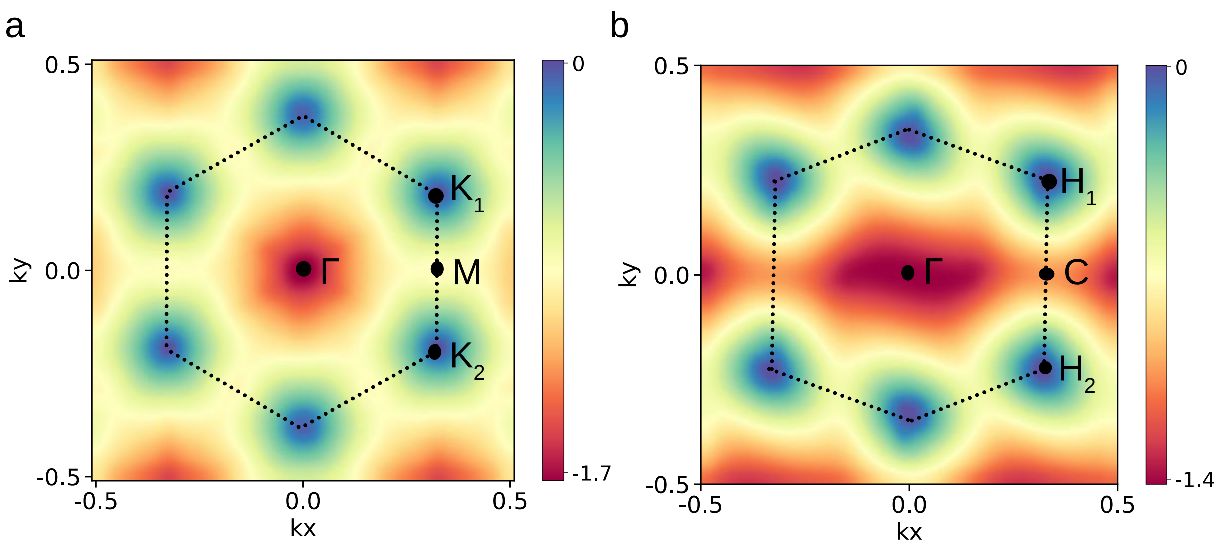

Figure S5 | 2D band structure. Left panel: the highest valence band of TBG without heterostrain. Right panel: the highest valence band of TBG with 1.3% heterostrain. The dashed frames indicate the Brillouin zone while the black points indicate the high-symmetry k points.

Supplementary Note 5: Simulation on DOS of heterostrained TBG

The density of the states can be calculated by the expression:

$$DOS(E) = \frac{1}{\pi}\sum_{n,\vec{k}} \frac{\gamma}{(E-E_{n\vec{k}})^2+\gamma^2} \tag{6}$$

where $E_{n,\vec{k}}$ is the eigenvalue of Bloch state based on the tight binding model, and $\gamma = 0.03\ eV$ is the broadening factor due to the impurities and disorder.

Supplementary Note 6: Simulation on dynamic conductivity of heterostrained TBG

The frequency-dependent optical conductivity tensor, expressed by the Kubo-Greenwood formula in the bases of Bloch states can be formulated as:[11,12]

$$\sigma_{\alpha\beta}(\omega) = \frac{-ie^2\hbar}{N_k S m_0^2} \sum_{m,n,\vec{k}} \left(\frac{f(E_{m\vec{k}}) - f(E_{n\vec{k}})}{E_{m\vec{k}} - E_{n\vec{k}}} \right) \frac{\langle m\vec{k}|p_\alpha|n\vec{k}\rangle\langle n\vec{k}|p_\beta|m\vec{k}\rangle}{\hbar\omega + E_{m\vec{k}} - E_{n\vec{k}} + i\gamma} \quad (7)$$

where p_α is the momentum operator along the α direction, N_k is the total sampling number of k points, S is the area of the unit cell, e is the electron charge, m_0 is the electron mass, and γ is the broadening factor. $f(E_{m\vec{k}})$ is the Fermi-Dirac distribution:

$$f(E_{m\vec{k}}) = \frac{1}{exp\left(\frac{E_{m\vec{k}} - \mu}{k_B T}\right) + 1} \quad (8)$$

where μ and k_B are the Fermi level and Boltzmann constant, respectively. In the calculation, we set the temperature $T = 300\ K$ and $\mu = 0.1\ eV$. We construct the Bloch states $|m\vec{k}\rangle$ using the tight binding basis:[13]

$$|m\vec{k}\rangle = \sum_{\vec{R}\mu\alpha} C_{\mu\alpha}^{m\vec{k}} e^{i\vec{k}\cdot(\vec{R}+\vec{r}_\mu)} |\mu\alpha, \vec{R}\rangle \quad (9)$$

where index μ labels the atoms in the primitive cell, α labels the orbitals on the given atom μ, R are the lattice vectors for the different unit cells, and $|\mu\alpha, \vec{R}\rangle$ is the tight-binding orbital. Usually, we use the atomic P_z orbital in graphene system. Since we only consider the single-orbital tight-binding model, thus the index α can be neglected. By solving the tight-binding eigen equations:

$$H_{\vec{k}} C^{m\vec{k}} = E_{m\vec{k}} C^{m\vec{k}} \quad (10)$$

we have the eigenenergy $E_{m\vec{k}}$ and eigenvector $C^{m\vec{k}}$. $C^{m\vec{k}}$ is the vector of the coefficient $C_\mu^{m\vec{k}}$. With the coefficient $C_\mu^{m\vec{k}}$, we have the constructed Bloch states, and the momentum matrix element $\langle m\vec{k}|p_\alpha|n\vec{k}\rangle$ can be calculated.[13]

Supplementary Note 7: Heterostrain-enabled optical transition between saddle points

The forbidden transition from S2 to S1 at point M between highest valence band and lowest conduction band in pristine TBG is due to the symmetry protection: $\Sigma^{-1}H\Sigma = -H^{*}$, where Σ is the 4×4 matrix defined as:

$$\Sigma = \begin{pmatrix} 0 & \sigma_x \\ -\sigma_x & 0 \end{pmatrix},\ \sigma_x = \begin{pmatrix} 0 & 1 \\ 1 & 0 \end{pmatrix} \tag{11}$$

However, it is not the case in the heterostrained TBG. Basically, the stain-induced gauge potential breaks the certain symmetry in the pristine TBG, and gives out a non-zero optical matrix elements at the saddle points. We start it from the effective low energy Hamiltonian[14] with heterostrain in one layer:

$$H_{\mathrm{eff}} = \begin{pmatrix} -\hbar v\vec{\sigma}\cdot\left(\vec{k} - \Delta K^{1} - \vec{A}\right) & U^{\dagger} \\ U & -\hbar v\vec{\sigma}\cdot\left(\vec{k} - \Delta K^{2}\right) \end{pmatrix} \tag{12}$$

where ΔK^{1} and ΔK^{2} are the wave vector shifts of Dirac cones by rotation in the first layer and second layer, respectively. U is the interlayer hopping matrix, $\vec{A}$ is the gauge potential induced by heterostrain:[15]

$$\vec{A} = \frac{\beta}{2a_0}(\epsilon_{11} - \epsilon_{22}, -2\epsilon_{12}) \tag{13}$$

where $(\epsilon_{11}, \epsilon_{22}, \epsilon_{12})$ are the elements of the strain tensor. One can easily check that the symmetry transformation is broken with strain induced gauge potential:

$$\Sigma^{-1}H_{eff}\Sigma \neq -H_{eff}^{*} \tag{14}$$

And the relation between S3 and S4 at saddle point C is also broken: $|\psi_{S3}\rangle \neq |\Sigma\psi_{S4}^{*}\rangle$, thus the optical matrix element $\langle\psi_{S3}|p_x\,|\psi_{S4}\rangle$ from S4 to S3 turns out to be nonzero according to the discussion in Ref. S12.[14]

Reference

S1. Qiu, U., Finlayson, G. D. & Qiu, G. Contrast maximizing and brightness preserving color to grayscale image conversion. in *CGIV 2008 Final Program and Proceedings* 347–351 (2008).

2. Kumar, T. & Verma, K. A Theory Based on Conversion of RGB image to Gray image. *Int. J. Comput. Appl.* **7**, 5–12 (2010).

3. Nair, R. R. *et al.* Fine structure constant defines visual transparency of graphene. *Science (80-.).* **320**, 1308 (2008).

4. Ferrari, A. C. *et al.* Raman spectrum of graphene and graphene layers. *Phys. Rev. Lett.* **97**, 187401 (2006).

5. Neubeck, S. *et al.* Direct determination of the crystallographic orientation of graphene edges by atomic resolution imaging. *Appl. Phys. Lett.* **97**, 0533110 (2010).

6. Gonzalez-Arraga, L. A., Lado, J. L., Guinea, F. & San-Jose, P. Electrically Controllable Magnetism in Twisted Bilayer Graphene. *Phys. Rev. Lett.* **119**, 1–6 (2017).

7. Lopes Dos Santos, J. M. B., Peres, N. M. R. & Castro Neto, A. H. Graphene bilayer with a twist: Electronic structure. *Phys. Rev. Lett.* **99**, 19–22 (2007).

8. Lopes Dos Santos, J. M. B., Peres, N. M. R. & Castro Neto, A. H. Continuum model of the twisted graphene bilayer. *Phys. Rev. B* **86**, 1–12 (2012).

9. Nam, N. N. T. & Koshino, M. Lattice relaxation and energy band modulation in twisted bilayer graphene. *Phys. Rev. B* **96**, 075311 (2017).

10. Yoo, H. *et al.* Atomic and electronic reconstruction at the van der Waals interface in twisted bilayer graphene. *Nat. Mater.* **18**, 448–453 (2019).

11. Lee, C. C., Lee, Y. T., Fukuda, M. & Ozaki, T. Tight-binding calculations of optical matrix elements for conductivity using nonorthogonal atomic orbitals: Anomalous Hall conductivity in bcc Fe. *Phys. Rev. B* **98**, 1–8 (2018).

12. Moon, P., Son, Y. W. & Koshino, M. Optical absorption of twisted bilayer graphene with interlayer potential asymmetry. *Phys. Rev. B* **90**, 1–10 (2014).

13. Pedersen, T. G., Pedersen, K. & Brun Kriestensen, T. Optical matrix elements in tight-binding calculations. *Phys. Rev. B* **63**, 0–3 (2001).

14. Moon, P. & Koshino, M. Optical absorption in twisted bilayer graphene. *Phys. Rev. B* **87**, 205404 (2013).

15. De Juan, F., Sturla, M. & Vozmediano, M. A. H. Space dependent Fermi velocity in strained graphene. *Phys. Rev. Lett.* **108**, 1–5 (2012).
